# Supplementary material for: Application of Three-Dimensional Culture Method in the Cardiac Conduction System Research
Source: Methods Protoc. 2022 Jun 14;5(3):50. doi: 10.3390/mps5030050 (PMC9227420; doi:10.3390/mps5030050)
Supplement: Supplementary file 1 [file mps-05-00050-s001.zip › mps-1709087-supplementary-proof done/Suppl. Material/Supplementary Material.pdf]

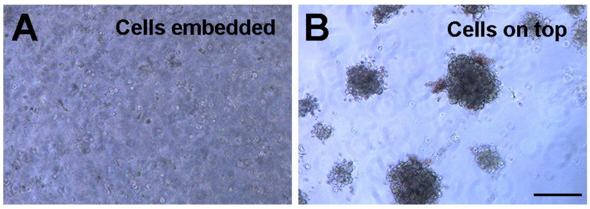

Figure S1. Examples of cells embedded in the matrix (**A**) and cells on the top of the matrix cultured for 5 days in Cultrex BME (**B**). Note the absence of spheroid formation in cells embedded in the matrix method. Bar = 100 μm.

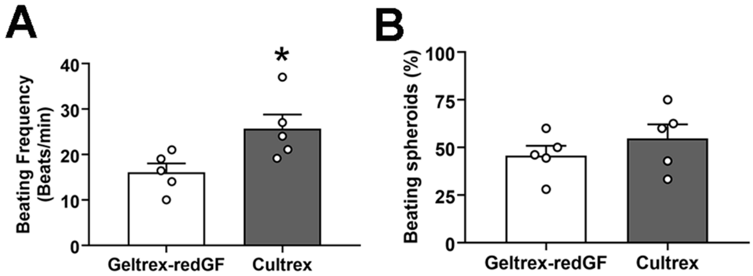

Figure S2: (**A**) Ventricular cells grown in Geltrex BME matrix with reduced growth factors (Geltrex-redGF) revealed significantly lower beating frequencies when compared to cells grown in regular Cultrex BME (without reduced growth factors) after day 8 in culture, (**B**) Percentage of beating spheroid cell bodies did not differ significantly between cells grown in Geltred-redGF and Cultrex BME matrices, N = 5 independent experiments, each bar represents mean ± SEM. * $p < 0.05$, Student's unpaired *t*-test.
