# Supplementary figures and images for: Application of Three-Dimensional Culture Method in the Cardiac Conduction System Research
Source: Methods Protoc. 2022 Jun 14;5(3):50. doi: 10.3390/mps5030050 (PMC9227420; doi:10.3390/mps5030050)

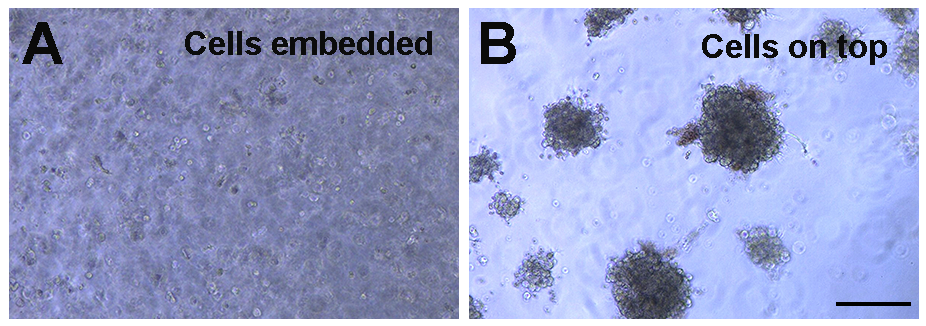

Supplement: Supplementary file 1 [file mps-05-00050-s001.zip › mps-1709087-supplementary-proof done/Suppl. Material/Figure S1.tif]

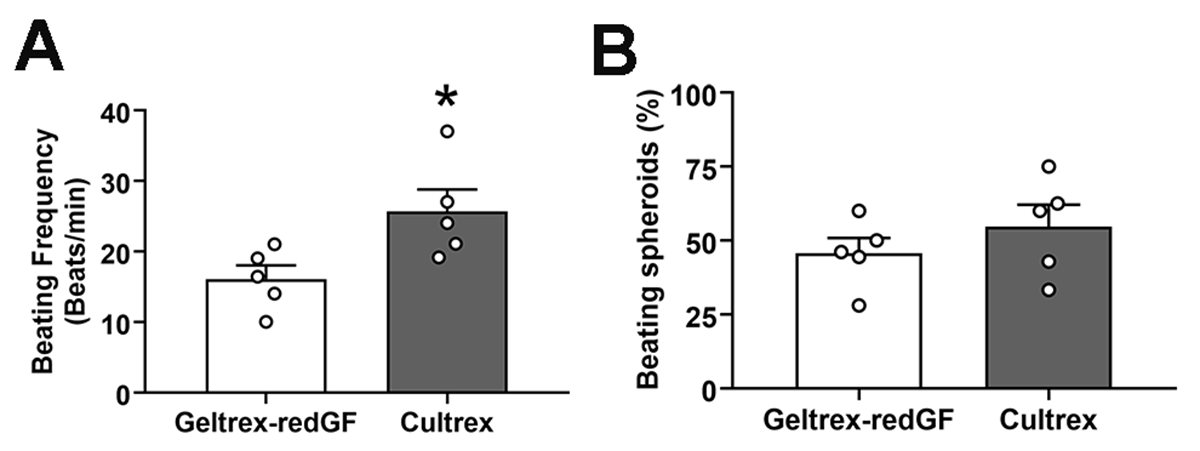

Supplement: Supplementary file 1 [file mps-05-00050-s001.zip › mps-1709087-supplementary-proof done/Suppl. Material/Figure S2.tif]
